# Use of Transcranial Magnetic Stimulation for Depression

**DOI:** 10.7759/cureus.4736

**Published:** 2019-05-23

**Authors:** Sukaina Rizvi, Ali M Khan

**Affiliations:** 1 Psychiatry, Manhattan Psychiatric Center, Manhattan, USA; 2 Psychiatry, University of Texas Rio Grande Valley, Harlingen, USA

**Keywords:** tms, depression, neuromodulation

## Abstract

Transcranial magnetic stimulation (TMS), a research tool with various effects on brain cells, can depolarize cerebral neurons noninvasively. This method offers temporal and spatial resolution and can be combined with other neurocognitive and neuro-experimental techniques. Prefrontal TMS therapy repeated daily for four to six weeks is a neuromodulation technique approved by the US Food and Drug Administration for the treatment of major depressive disorder (MDD) in patients resistant to medications. This technique utilizes electromagnetic induction to excite neuronal cells. Several recent studies have enhanced our understanding of this novel treatment intervention. This report reviews recent studies on the mechanism of action, patient eligibility, effectiveness, and safety of TMS in treating depression.

## Introduction and background

Depression is a serious mental health condition characterized by a persistently depressed mood and diminished interest in activities and can lead to severe functional impairment in daily life. In diagnosing depression, the fifth edition of the Diagnostic and Statistical Manual of Mental Disorders requires five or more symptoms to be present within a two-week period, with one of these symptoms being a depressed mood or anhedonia (loss of interest or pleasure). Secondary symptoms of major depression include changes in appetite and/or weight changes, sleep difficulties (insomnia or hypersomnia), psychomotor agitation or retardation, fatigue or loss of energy, diminished ability to think or concentrate, feelings of worthlessness or excessive guilt, and suicidal ideation. The lifetime prevalence of depression in the general population has been estimated to be 10% [1-2].

Major depressive disorder (MDD) is a highly prevalent psychiatric disorder with significant morbidity and mortality. Psychotherapy, medications, and their combination are often insufficient to control the symptoms of MDD, resulting in treatment-resistant depression (TRD). This leads to poor functional outcomes in patients, including increased unemployment, suicidal ideation, substance abuse, and unstable relationships. Transcranial magnetic stimulation (TMS) has been successful in treating patients with TRD. A literature review reported that the response rates to TMS range between 50% and 55%, and the remission rates range between 30% and 35% in patients with major depression [3]. The dorsolateral prefrontal cortex (DLPFC) is the part of the brain that has been reported dysregulated in patients with major depression, resulting in symptoms consistent with this condition depression [4]. TMS targets the pathophysiology of depression, using high-frequency electromagnetic induction over the left DLPFC to effectively treat the behavioral dysregulation in patients with major depression.

## Review

TMS is an advanced treatment modality for patients with TRD, in which brief magnetic pulses are applied to the brain, leading to cortical stimulation. Repetitive TMS (rTMS) consists of the delivery of pulses in a repetitive fashion. TMS is a non-invasive technique, as it does not require craniotomy or seizure induction to stimulate nerve cells [5]. Rather, TMS interconverts electrical and magnetic energy to induce electromagnetic phenomena. An electromagnetic coil is placed on the scalp, and an effective pulsatile magnetic field that depolarizes cortical neurons is generated for a brief duration of time. This forceful magnetic field is generated on the surface of the scalp, without any intrusion into the skin, muscles or bones, making its electrical stimulation electrodeless [5-6]. Levels of excitability of neuronal stimulation can be regulated, either by high (10 to 20 Hz) or low (1 Hz) frequency. The generated magnetic field is about 1.5 Tesla, similar in strength to that of magnetic resonance imaging [7]. Patients being treated with TMS undergo five daily treatment sessions over three to six weeks, resulting in a total of 20 to 30 sessions during treatment.

High-frequency stimulation of the left DLPFC alleviates depressive symptoms, whereas low-frequency cortical stimulation of the right DLPFC helps to relieve the symptoms of both depression and anxiety. This was validated by a case series in which the effects of TMS on either side of the prefrontal cortex were evaluated in 16 healthy individuals aged 24 to 75 years. Patients who underwent stimulation of the left DLPFC showed a drop in salivary cortisol level in the morning. However, the results of this study were limited due to the small sample size and limited number of TMS sessions [6]. Many randomized clinical trials have shown that daily TMS of the left prefrontal cortex was effective in treating depressive mood symptoms, with remission rates of 30% to 40% of cases. Similar to antidepressants, TMS resulted in sustained improvement in mood symptoms but with fewer side effects [5,8].

Another randomized multicenter trial showed that this non-invasive treatment method had significant antidepressant effects with remission rates four-fold higher than placebo. A multicenter observational study involving 307 patients in a clinical setting showed that the depression severity scale decreased after treatment with TMS. Moreover, therapeutic responses were maintained in patients treated with TMS [5,9-10]. TMS was found to significantly improve all symptoms of depression on the Hamilton depression rating scale [11]. Another randomized, controlled, multicenter, multiphase trial, performed at 23 sites worldwide, assessed the effects of TMS in patients diagnosed with MDD and free of the side effects of any antidepressant. This trial showed that patients who underwent TMS demonstrated clinically and statistically significant improvements on the Montgomery-Asberg Depression Rating Scale (MASDR) after four weeks; however, this outcome was similar to that observed in patients randomized to placebo [5,12]. Another study provided descriptive evidence of the long-term therapeutic benefits of TMS and maintenance of benefits with post-TMS antidepressant therapy [13].

The neurobiological phenomena underlying the effectiveness of TMS as an antidepressant are not well understood, although studies have suggested a correlation between cerebral metabolic activity and TMS effectiveness. This was validated by the results of TMS treatment in non-responders who exhibited hypoperfusion in the frontal cortex. A pilot study involving 15 medication-resistant patients demonstrated an increase in cerebral blood volume during TMS. Blood flow was measured at the beginning and end of each TMS session, with the frontal hemoglobin concentration (fHbC) found to be associated with treatment outcomes, in that increased fHbC during magnetic induction was inversely related to MASDR scores. This study was limited due to the small sample size and because cerebral blood flow was not measured in the middle of treatment with TMS [14-15]. Neuronal physiology that responds to TMS is of central importance, as repetitive stimulations increase synaptic plasticity, causing it to last longer even after stimulation ceases. This result is contrary to the decrease in synaptic strength observed in depression and may be attributed to the fact that fast rTMS (> 10 Hz) results in neuronal excitation while slow rTMS (< 1 Hz) has the reverse effects. Another study reported that daily sessions of TMS over the prefrontal cortex were found to increase cortical activity and reduce activity in distant areas; moreover, cerebral blood flow at the stimulation site decreased with increasing coil distance from the scalp to the outer cortex [16]. As recommended, neuromodulation therapy via TMS is usually performed after failure of an antidepressant trial, with or without psychotherapy.

Magnetoencephalography has provided insights into the possible role of neuronal circuits in TMS-induced improvements in depression. This method found that clinical benefits of TMS in depression are due, at least in part, to the functional connectivity of the left DLPFC with the limbic system [17-19].

TMS is regarded as an effective and safe stimulation technique. However, it has a few side effects that might affect treatment compliance. Commonly reported adverse effects include transitory and/or recurrent headaches that respond to over the counter analgesics, a tingling sensation on the scalp and face, and ipsilateral lacrimation. Seizure activity is rare, and one patient was reported to develop trigeminal autonomic cephalalgia after the administration of rTMS for refractory depression [20].

## Conclusions

The clinical efficacy of TMS as an antidepressant has been well established. TMS is an innovative and promising treatment modality for patients with TRD. Patient compliance, however, may be affected, as TMS requires frequent visits to the clinic. Neuromodulation through TMS may induce neurogenesis. Further research is required to explore its therapeutic implementation and limitations, providing more effective treatment of patients with TRD.

## References

[1] Kessing LV (2007). Epidemiology of subtypes of depression. Acta Psychiatr Scand Suppl.

[2] Tolentino JC, Schmidt SL (2018). DSM-5 criteria and depression severity: implications for clinical practice. Front Psychiatry.

[3] Reddy MS, Vijay MS (2017). Repetitive transcranial magnetic stimulation for depression: state of the art. Indian J Psychol Med.

[4] Janicak PG, Dokucu ME (2015). Transcranial magnetic stimulation for the treatment of major depression. Neuropsychiatr Dis Treat.

[5] George MS, Taylor JJ, Short EB (2013). The expanding evidence base for rTMS treatment of depression. Curr Opin Psychiatry.

[6] Ishida FA, Kobayashi A, Hu A, Yamaguchi T, Watahiki H, Kobayashi H (2018). A case study: the effect of transcranial magnetic stimulation (TMS) on stress levels, quality of sleep, and the autonomic nervous system. Adv Clin Transl Res.

[7] Perera T, George MS, Grammer G, Janicak PG, Pascual-Leone A, Wirecki TS (2016). The Clinical TMS Society consensus review and treatment recommendations for TMS therapy for major depressive disorder. Brain Stimul.

[8] Roth Y, Amir A, Levkovitz Y, Zangen A (2007). Three-dimensional distribution of the electric field induced in the brain by transcranial magnetic stimulation using figure-8 and deep H-coils. J Clin Neurophysiol.

[9] Demitrack MA, Thase ME (2009). Clinical significance of transcranial magnetic stimulation (TMS) in the treatment of pharmacoresistant depression: synthesis of recent data. Psychopharmacol Bull.

[10] Connolly KR, Helmer A, Cristancho MA, Cristancho P, O’Reardon JP (2012). Effectiveness of transcranial magnetic stimulation in clinical practice post-FDA approval in the United States: results observed with the first 100 consecutive cases of depression at an academic medical center. J Clin Psychiatry.

[11] May T, Pridmore S (2019). Impact of transcranial magnetic stimulation on the symptom profile of major depressive episode. Australas Psychiatry.

[12] O’Reardon JP, Solvason HB, Janicak PG (2007). Efficacy and safety of transcranial magnetic stimulation in the acute treatment of major depression: a multisite randomized controlled trial. Biol Psychiatry.

[13] Mantovani A, Pavlicova M, Avery D (2012). Long term efficacy of repeated daily prefrontal transcranial magnetic stimulation (TMS) in treatment-resistant depression. Depress Anxiety.

[14] Shinba T, Kariya N, Matsuda S, Matsuda H, Obara Y (2018). Increase of frontal cerebral blood volume during transcranial magnetic stimulation in depression is related to treatment effectiveness: a pilot study with near-infrared spectroscopy. Psychiatry Clin Neurosci.

[15] Richieri R, Boyer L, Farisse J, Colavolpe C, Mundler O, Lancon C, Guedj E (2011). Predictive value of brain perfusion SPECT for rTMS response in pharmacoresistant depression. Eur J Nucl Med Mol imaging.

[16] Nahas Z, Teneback CC, Kozel A (2001). Brain effects of TMS delivered over prefrontal cortex in depressed adults: role of stimulation frequency and coil-cortex distance. J Neuropsychiatry Clin Neurosci.

[17] Wassermann EM, Wedegaertner FR, Ziemann U, George MS, Chen R (1998). Crossed reduction of human motor cortex excitability by 1-Hz transcranial magnetic stimulation. Neurosci Lett.

[18] Post RM, Kimbrell TA, McCann UD, Dunn RT, Osuch EA, Speer AM, Weiss SR (1999). Repetitive transcranial magnetic stimulation as a neuropsychiatric tool: present status and future potential. J ECT.

[19] Fox MD, Buckner RL, White MP, Greicius MD, Pascual-Leone A (2012). Efficacy of transcranial magnetic stimulation targets for depression is related to intrinsic functional connectivity with the subgenual cingulate. Biol Psychiatry.

[20] Durmaz O, Ateş MA, Şenol MG (2015). Repetitive transcranial magnetic stimulation (rTMS)-induced trigeminal autonomic cephalalgia. Noro Psikiyatr Ars.

